# Genomic Assortative Mating in Marriages in the United States

**DOI:** 10.1371/journal.pone.0112322

**Published:** 2014-11-10

**Authors:** Guang Guo, Lin Wang, Hexuan Liu, Thomas Randall

**Affiliations:** 1 Department of Sociology, the University of North Carolina at Chapel Hill, Chapel Hill, North Carolina, the United States of America; 2 Carolina Population Center, the University of North Carolina at Chapel Hill, Chapel Hill, North Carolina, the United States of America; 3 Carolina Center for Genome Sciences, the University of North Carolina at Chapel Hill, Chapel Hill, North Carolina, the United States of America; 4 Center for Child and Family Policy, Duke University, Durham, North Carolina, the United States of America; 5 National Institute of Environmental Health Sciences, Research Triangle Park, North Carolina, the United States of America; University of Utah, United States of America

## Abstract

Assortative mating in phenotype in human marriages has been widely observed. Using genome-wide genotype data from the Framingham Heart study (FHS; number of married couples = 989) and Health Retirement Survey (HRS; number of married couples = 3,474), this study investigates genomic assortative mating in human marriages. Two types of genomic marital correlations are calculated. The first is a correlation specific to a single married couple “averaged” over all available autosomal single-nucleotide polymorphism (SNPs). In FHS, the average married-couple correlation is 0.0018 with p = 3×10^−5^; in HRS, it is 0.0017 with p = 7.13×10^−13^. The marital correlation among the positively assorting SNPs is 0.001 (p = .0043) in FHS and 0.015 (p = 1.66×10^−24^) in HRS. The sizes of these estimates in FHS and HRS are consistent with what are suggested by the distribution of the allelic combination. The study also estimated SNP-specific correlation “averaged” over all married couples. Suggestive evidence is reported. Future studies need to consider a more general form of genomic assortment, in which different allelic forms in homologous genes and non-homologous genes result in the same phenotype.

## Introduction

Assortative mating refers to a systematic departure from random mating. Positive assortative mating or homogamy occurs when mating individuals have similar traits, and negative assortative mating or heterogamy occurs when mating individuals have dissimilar traits. Human assortative mating in phenotype has been investigated for more than a century. In 1903, Pearson and colleagues report that the correlations in height, the span of arms, and the length of left forearm between husband and wife are 0.28, 0.20, and 0.20, respectively, drawing on extensive family records of 1,000 husband-wife pairs. Since Pearson’s work, marriage partners have been shown to assort on a wide range of traits including race and ethnicity, age, propinquity in geography, religious belief, socio-economic status (such as educational attainment, occupation, and income), cognitive ability, anthropometric measures (such as weight, height, skin pigmentation, and other related measures), personality characteristics, mental and psychiatric conditions, and political attitudes (e.g., [1], [2]–[13]).

**Figure 1:**
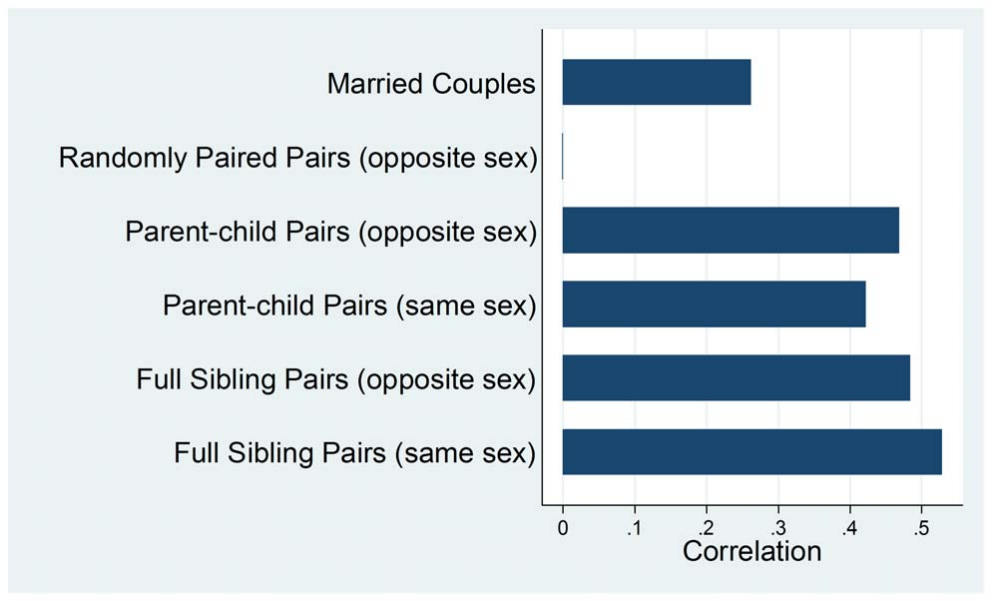
FHS data – the correlation of height (standardized within each sex) for married couples (N of couples = 989), opposite-sex random pairs from permuted individuals in FHS (N = 200,000), opposite-sex parent-child pairs (N = 3,447), same-sex parent-child pairs (N = 3,511), opposite-sex full sibling pairs (N = 2,815), and same-sex full sibling pairs (N = 2,898).

If marriages are assorted to a degree by individual traits and if these traits are to a degree associated with genetic variation, it would be reasonable to hypothesize a degree of genetic assortment in human marriages. As an illustrative example, the heritability of human height is about 0.80 in developed countries [14]. Recent genome-wide association studies (GWAS) have found at least 180 independent regions of the genome that are associated with height [15]–[19]. Figure 1 shows the correlation of height for different types of pairs using data from the Framingham Heart Study (FHS), with height standardized within each sex. The data show a correlation of about one half for same-sex as well as opposite-sex full-sibling pairs and parent-child pairs. The correlation for randomly paired individuals is essentially zero. The correlation for married couples in FHS after adjusting for population structure is about 0.27. This marital assortment in height likely has a major genetic component.

Genetic assortative mating may have reproductive consequences. Thiessen and Gregg [6] hypothesize that positive assortative mating outside nuclear families increases the genetic relatedness within a family, which in turn increases inclusive fitness without an extra reproductive effort. Lewontin [3] suggests that human assortative mating may play a major role in redistributing genes in contemporary times, particularly because selection through death has largely been replaced by selection through birth due to sharply-reduced mortality. If mating partners do share similar genetic variants related to, for example, obesity or psychiatric conditions, the impact of these genetic variants on the couples’ offspring may be compounded. The role of genetic assortative mating may evolve with social trends. For example, college-educated Americans are increasingly more likely to marry each other rather than those with less education in comparison to a half-century ago [20]. This educational assortative mating reinforces a growing social divide between those with very low levels of education and those with more education, magnifying social class differences. This growing social divide could be partially genetic because of assortative mating.

Pearson [21] conjectures that, on average, a husband and wife are more alike than first cousins, whose coefficient of genetic relatedness is 0.125 and probably as much alike as uncle and niece, whose coefficient of genetic relatedness is 0.25, apparently basing the conjectures on the correlation findings over anthropometric measures. Pearson compares human homogamy to self-fertilization in plants; nevertheless, he realizes that human homogamy may have any degree of intensity and may be restricted to certain traits because genetic assortment can only be accomplished through phenotype.

In this project, we assess the extent to which marriage partners assort genetically using genome-wide genotype (GWAS) data from two independent studies in the United States for replication: 989 married couples in the Framingham Heart Study [22] (FHS) and 3,474 married couples in the Health and Retirement Survey (HRS). We carry out three sets of analyses: the first analysis uses 989 married couples and 287,294 SNPs in FHS; the second uses 3,474 couples and 66,526 SNPs (these 66,526 SNPs are common to both genotyping platforms used in the FHS and HRS studies); and the third analysis repeats the FHS analysis using the same 66,526 SNPs that are commonly available in FHS and HRS.

This analysis focuses on genomic assortative mating beyond race and ethnicity. It is well-known that marriages in the United States assort on race and ethnicity (e.g., [9], [23]). To estimate genetic correlation within married couples net of race and ethnicity, population stratification must be controlled. In our analysis, population stratification is controlled directly in the regression models that estimate genomic assortment.

To estimate genetic assortative mating at the genomic level, we calculate two types of genome-wide marital correlations. The first is a correlation specific to a single married couple (couple correlation) “averaged” over all available autosomal SNPs. For FHS, this calculation yields 989 correlation estimates, one for each married couple averaged over 287,294 SNPs. Married-couple correlations provide a global or genomic estimate of the correlation averaged over the human genome. Such a measure is possible and attempted in this project because assortative mating may occur over a number of human traits. Negative genomic assortment is a potential complication that may cancel negative and positive genomic assortment within a single married couple. Although assortative mating is generally considered positive, negative assortment or that opposites attract is likely to be present [1], . To address this issue, we estimate two additional correlations for each married couple. One is based on about half of the 287,294 SNPs that assort more positively and the other is based on the other half that assort more negatively.

The second marital correlation is a SNP correlation “averaged” over all married couples. For FHS, the SNP correlation analysis yields 287,294 correlations, one for each SNP averaged over 989 married couples. The analysis of couple correlations is quite distinct from GWAS studies. It is concerned with genetic similar within a couple averaged over the genome; it is also far more computationally demanding than a GWAS analysis. The analysis of SNP correlations appears to resemble a GWAS analysis: a GWAS study examines each SNP’s association with a single phenotype in a collection of individuals and a SNP-correlation analysis estimates the average correlation over a collection of married couples with respect to a SNP. However, an important difference between the two is that married couples may assort on different phenotypes and thus assort at different genetic loci, which makes it more difficult for the analysis of SNP correlations to produce reliable estimates than a GWAS analysis.

Recent work by Domingue et al. [24] provides an estimate of genome-wide genetic similarity and an estimate of educational similarity within spousal pairs, concluding that the spousal genetic similarity over the genome is about one third or one fourth of the spousal educational similarity. Although using the same two data sources of FHS and HRS, our analysis was independently performed and reveals a number of additional insights. We use a different measure of spousal genomic similarity, calculate additional two measures of couple correlation for each married couple, and estimate SNP-correlations.

## Results

**Figure 2:**
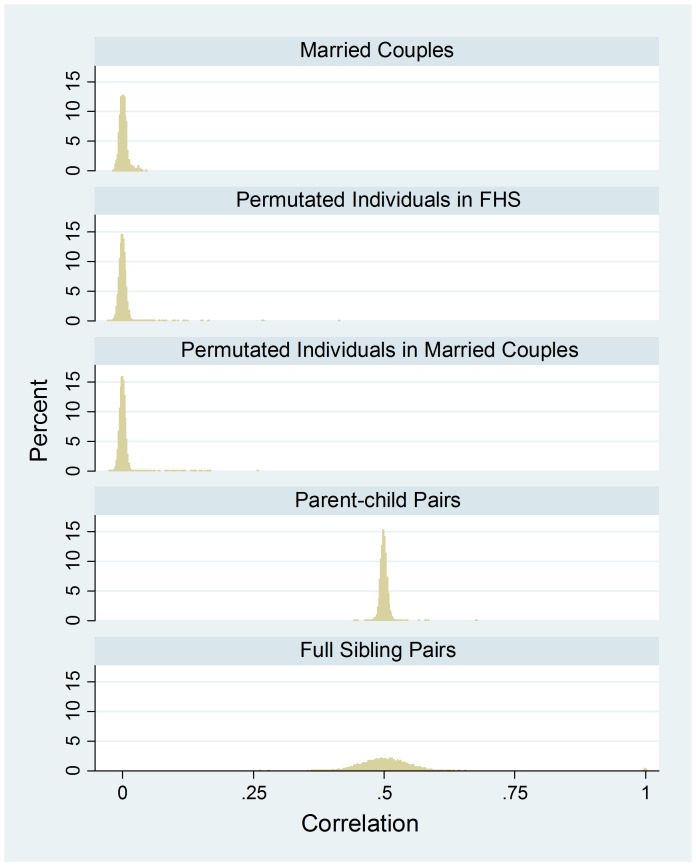
FHS data – the empirical density distribution of couple correlation for married-couples (N = 989), opposite-sex random pairs from permuted individuals in FHS (N = 200,000), opposite-sex random pairs from permuted individuals among married couples (N = 246,870), parent-child pairs (N = 6,958), and full sibling pairs (N = 5,713), with each mixed-model regression estimating a within-a-single-pair correlation “averaged” over 287,294 SNPs.

Figure 2 shows the FHS distribution of couple correlation for married couples (N = 989), opposite-sex random pairs from permuted individuals in FHS (N = 200,000), opposite-sex random pairs from permuted individuals among married couples (N = 246,870), full-sibling pairs (N = 5,713), and parent-child pairs (N = 6,958). After controlling for population admixture, the married-couple correlations average 0.0018 relative to the average of randomly paired individuals (Panel 1 of Table 1). The correlation is highly significant according to both permutation tests. In contrast, the pair-specific correlations for full-sibling pairs and parent-child pairs are both centered on 0.50 with a mean of 0.503 (SD = 0.053) and 0.499 (SD = 0.007), respectively. As expected, the standard deviation of the parent-child pairs is much smaller than that of the full siblings.

**Table 1:** FHS and HRS data – Two permutation tests for married-couple correlations within “negative” and “positive” SNPs: (1) permuted individuals in 989 (FHS) and 3,474 (HRS) married couples, respectively, and (2) permuted all individuals in FHS.

FHS data			
		Permuted individuals in 989married couples	Permuted all individualsin the FHS
All SNPs	Mean difference in correlation: (Married couplesminus random pairs)	0.0018	0.0018
	Average p-values	0.00003	0.0001
	Proportion of p-values <0.05	99.98%	99.94%
Negative “half” of SNP combinations:20/02, 01/10, and 11	Mean difference in correlation: (Married couplesminus random pairs)	−0.000076	0.00036
	Average p-values	0.417	0.178
	Proportion of p-values <0.05	8.94%	36.14%
Positive “half” of SNP combinations:00,12/21, and 22	Mean difference in correlation: (Marriedcouples minus random pairs)	0.00095	0.0012
	Average p-values	0.0043	0.0088
	Proportion of p-values <0.05	98.14%	96.32%
**HRS data**			
		**Permuted individuals in 3,474** **married couples**	**Permuted all individuals** **in the HRS**
All SNPs	Mean difference in correlation: (Married couplesminus random pairs)	0.0017	0.0016
	Average p-values	7.13×10^−13^	8.39×10^−12^
	Proportion of p-values <0.05	100%	100%
Negative “half” of SNP combinations:20/02, 01/10, and 11	Mean difference in correlation: (Married couplesminus random pairs)	−0.0012	−0.0012
	Average p-values	0.0023	0.0016
	Proportion of p-values <0.05	99.2%	99.3%
Positive “half” of SNP combinations:00, 12/21, and 22	Mean difference in correlation: (Married couplesminus random pairs)	0.015	0.020
	Average p-values	1.66×10^−24^	7.75×10^−41^
	Proportion of p-values <0.05	100%	100%

**Figure 3:**
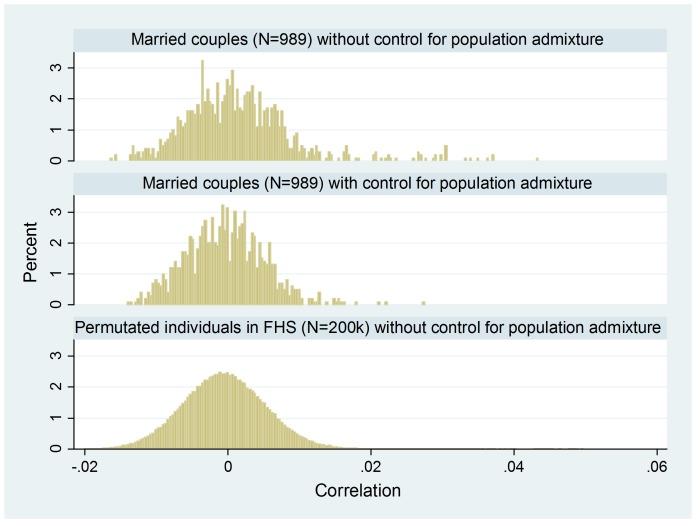
FHS data – the empirical density distribution of married-couple correlation over the 287,295 SNPs; (1) married couples (N = 989) without control for population admixture, (2) married couples (N = 989) with control for population admixture, and (3) opposite-sex random pairs from permuted individuals in FHS (N = 200,000). Panels (2) and (3) are the same as Panels (1) and (2) in Figure 2 and enlarged.

Figure 3 shows the effect of controlling for population admixture via adding seven main principal components in FHS. The figure presents two estimated distributions of married-couple correlation (Panels 1 and 2) and the distribution of pair correlations estimated from random pairs (Panel 3). The results in Panels 1 and 2 are without and with control for population admixture, respectively. Once population admixture is controlled, the couple correlations that are larger than 0.02 have vanished (Panel 2).

**Figure 4:**
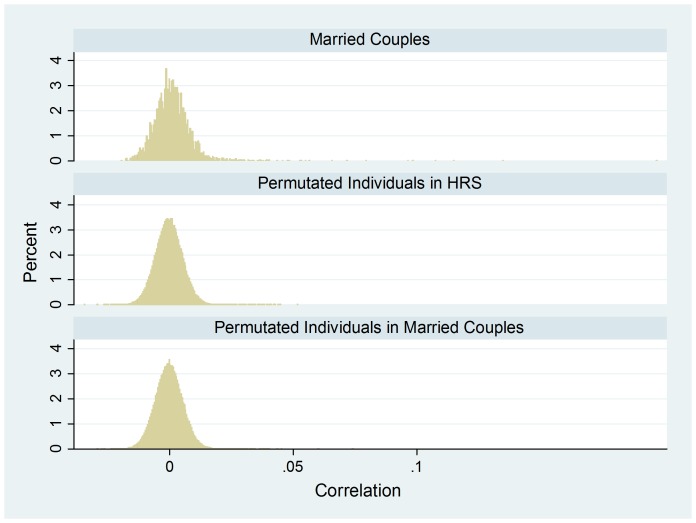
HRS data – the empirical density distribution of couple correlation for married-couples (N = 3,474), opposite-sex random pairs from permuted individuals (N = 200,000), and opposite-sex random pairs from permuted individuals among married couples (N = 200,000), with each mixed-model regression estimating a within-a-single-pair correlation “averaged” over 66,526 SNPs. These 66,526 SNPs are also available in FHS.

Figure 4 shows the HRS distribution of pair correlation, for married couples (N = 3,474), opposite-sex random pairs from permuted individuals in HRS (N = 200,000), and opposite-sex random pairs from permuted individuals among married couples (N = 200,000), with each mixed-model regression estimating a within-a-single-pair correlation “averaged” over the 66,526 SNPs. The results from the two permutation tests in Panel 2 of Table 1 suggest that averaged over the genome, married couples in HRS has a correlation of 0.0016–0.0017 relative to permuted random pairs. The results from both tests are highly significant. This HRS finding is similar to that from FHS.

Panel 1 of Table 2 presents FHS distribution of within-pair allelic combination for married couples, random pairs permuted among married couples, random pairs permuted among all FHS subjects, parent-child pairs, and full-sibling pairs. Large differences exist between genetically-related pairs (GRPs) and genetically non-related pairs (GNPs). Consistent with our hypothesis, GRPs tend to have a much higher percentage in allelic combinations of 22, 12 or 21, and 00 that contribute to positive assortment than GNPs. GNPs tend to have a much higher percentage than GRPs in allelic combinations of 02 or 20, 01 or 10, and 11 that contribute to negative assortment. Consistent with Figure 2, married couples exhibit an allelic distribution that is almost identical to those from the two sets of random pairs. However, a careful comparison reveals that married couples have slightly higher proportions of positive-assorting SNP combinations (22, 12 or 21, and 00) than those among the two types of random pairs, suggesting that the positive genomic correlation for married couples be slightly higher than that of random pairs. For the negatively assorting combinations (02 or 20, 10 or 01, and 11), the differences between married couples and random pairs are small and the directions are mixed. Compared with random pairs, married couples have a lower proportion in 02 or 20, and 10 or 01, but a higher proportion in 11, suggesting that the negative genomic correlation for married couples be zero or extremely small.

**Table 2:** FHS and HRS data – the observed distribution of within-pair allelic combination for different types of pairs [%(standard deviation)] for a total of 287,294 SNPs (FHS), 66,526 SNPs (HRS), and 66,526 SNPs (FHS common to those in HRS).

Panel 1: FHS					
Within-paircombination	MarriedCouplesN = 989	Random pairs permuted amongmarried Couples N = 246,870	Random pairs permuted amongall FHS subjects N = 200,000	Parent-childPairs N = 6,958	Full siblingPairs N = 5,713
02/20	6.457(0.21)	6.474(0.21)	6.457(0.22)	0.024(0.02)	1.606(0.30)
01/10	32.508(0.26)	32.560(0.25)	32.578(0.26)	22.661(0.23)	19.344(1.87)
11	12.725(0.22)	12.714(0.21)	12.737(0.21)	16.260(0.23)	19.560(1.23)
00	40.061(0.32)	40.021(0.32)	40.009(0.38)	48.190(0.32)	49.050(1.10)
12/21	7.069(0.14)	7.058(0.12)	7.050(0.12)	9.922(0.16)	6.690(0.60)
22	1.180(0.06)	1.173(0.05)	1.169(0.05)	2.943(0.12)	3.757(0.41)
Total	100%	100%	100%	100%	100%
**Panel 2: HRS**					
**Within-pair** **combination**	**Married** **Couples** **N = 3,474**	**Random pairs permuted among** **married Couples N = 200,000**	**Random pairs permuted among** **all FHS subjects N = 200,000**		
02/20	6.728(0.44)	7.360(1.24)	7.580(1.40)		
01/10	31.971(0.72)	32.688(1.26)	32.905(1.41)		
11	13.192(0.35)	12.956(0.60)	12.865(0.66)		
00	37.146(0.67)	36.470(1.22)	36.251(1.37)		
12/21	8.819(0.22)	8.583(0.43)	8.512(0.48)		
22	2.143(0.22)	1.943(0.33)	1.888(0.37)		
Total	100%	100%	100%		
**Panel 3: HRS for a total of 66,526 SNPs in FHS that are also available in HRS**					
**Within-pair** **combination**	**Married** **Couples** **N = 989**	**Random pairs permuted among** **married Couples N = 246,870**	**Random pairs permuted among** **all FHS subjects N = 200,000**	**Parent-child** **Pairs N = 6,958**	**Full sibling** **Pairs N = 5,713**
02/20	6.708(0.22)	6.729(0.23)	6.714(0.23)	0.027(0.03)	1.669(0.30)
01/10	33.459(0.29)	33.513(0.27)	33.524(0.28)	23.390(0.25)	19.948(1.88)
11	13.233(0.23)	13.224(0.23)	13.241(0.22)	16.815(0.25)	20.246(1.26)
00	38.037(0.36)	37.993(0.35)	37.988(0.40)	46.407(0.35)	47.300(1.12)
12/21	7.338(0.15)	7.327(0.14)	7.321(0.14)	10.308(0.18)	6.947(0.62)
22	1.224(0.07)	1.215(0.06)	1.212(0.06)	3.054(0.13)	3.901(0.42)
Total	100%	100%	100%	100%	100%

Panel 2 of Table 2 provides the observed HRS distribution of within-pair allelic combination for different types of pairs for the 66,526 SNPs. Comparing married couples against random pairs in HRS yields a similar pattern to that in FHS: the proportions of positively assorting allelic combinations in married couples are consistently higher than those in random pairs. These allelic data in HRS suggest that the “positive” half of the SNPs for married couples have a positive correlation while the negative correlation may be zero or extremely small. Comparing FHS and HRS, the proportion of positive assorting allelic combinations in married couples relative to random pairs appears considerably higher in HRS than in FHS, suggesting that the “positive” half of the SNPs for married couples in HRS have a larger positive correlation than those in FHS. These expectations are confirmed by regression findings.

**Figure 5:**
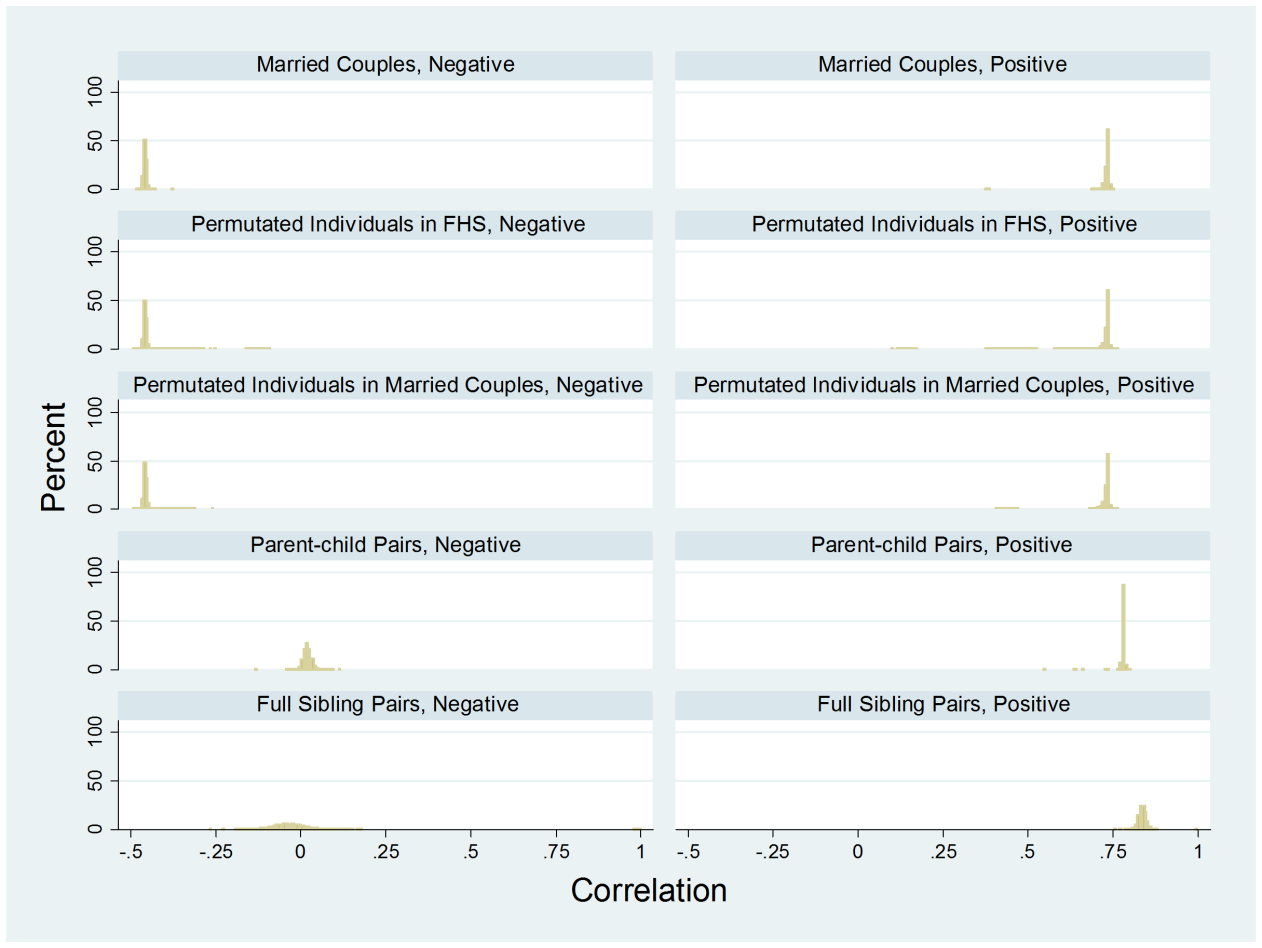
FHS data – the empirical density distribution of the “positive” and “negative” couple correlation, for married couples (N = 989), opposite-sex random pairs from permuted individuals in FHS (N = 200,000), opposite-sex random pairs from permuted individuals among married couples (N = 246,870), parent-child pairs (N = 6,958), and full sibling pairs (N = 5,713), with each mixed-model regression estimating the within a single-pair correlation “averaged” over about one half of the 287,294 SNPs.

Figure 5 provides the FHS empirical distribution of the “positive” and “negative” pair correlation, for married couples (N = 989), opposite-sex random pairs from permuted individuals in FHS (N = 200,000), and opposite-sex random pairs from permuted individuals among married couples (N = 246,870), with each mixed-model regression estimating the within a single-pair correlation “averaged” over about one half of the 287,294 SNPs.

The second half of Panel 1 of Table 1 shows the FHS results of two permutation tests for the married-couple correlations within the “negative” and “positive” SNPs. The two tests yield essentially identical findings. For the “negative” SNPs, the difference between the married-couple correlation and the random-pair correlation is small and statistically non-significant. In contrast, for the “positive” SNPs, the average of the married-couple correlation minus the random-pair correlation is about 0.001 and statistically significant according to the average p-values (0.0043 and 0.0088).

**Figure 6:**
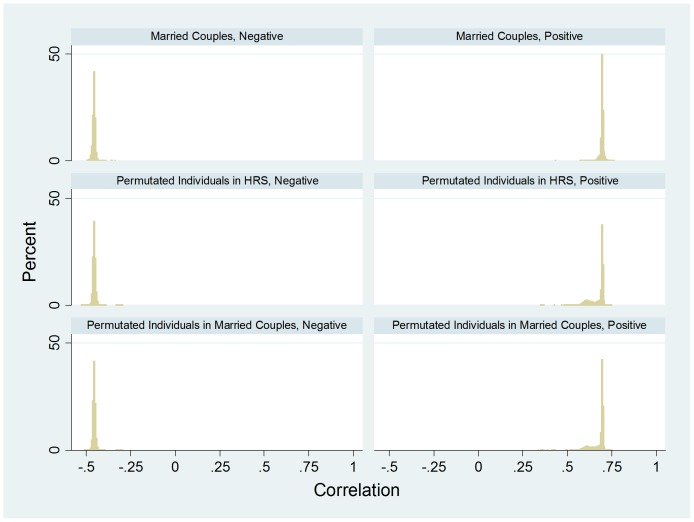
HRS data – the empirical density distribution of the “positive” and “negative” couple correlation for married couples (N = 3,474), opposite-sex random pairs from permuted individuals in the HRS (N = 200,000), and opposite-sex random pairs from permuted individuals among married couples (N = 200,000), with each mixed-model regression estimating the within a single-pair correlation “averaged” over about one half of the 66,526 SNPs.

Figure 6 presents the HRS distribution of the “positive” and “negative” pair-specific correlation, for married couples (N = 3,474), opposite-sex random pairs from permuted individuals in the HRS (N = 200,000), and opposite-sex random pairs from permuted individuals among married couples (N = 200,000), with each mixed-model regression estimating the within a single-pair correlation “averaged” over about one half of the 66,526 SNPs.

The second half of Panel 2 of Table 1 presents two permutation tests for HRS data – Two permutation tests for couple-specific correlations within “negative” and “positive” SNPs. Like in the FHS data, the two tests yield very similar findings. For the “negative” SNPs, on average, married couples have a small and statistically significant negative correlation (−0.0012, p = 0.0023; −0.0012, p = 0.0016). For the “positive” SNPs, on average married couples show a correlation of about 0.015 and 0.020, respectively, with extremely small p-values of 1.66×10^−24^ and 7.75×10^−41^.

**Figure 7:**
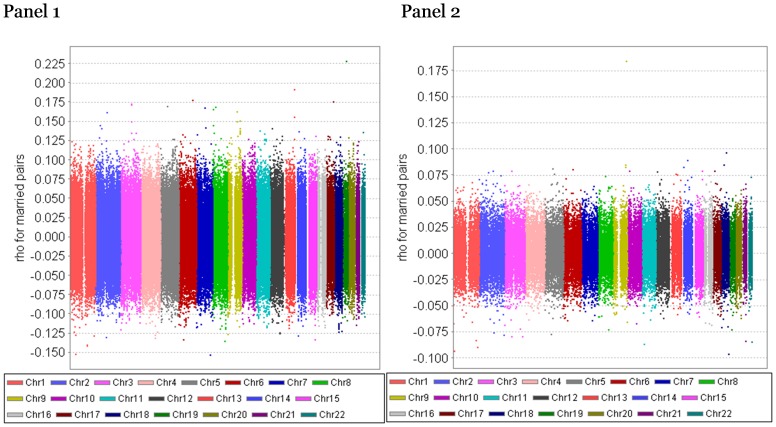
Panel 1: FHS data – genome-wide SNP-specific correlation for each of the 287,294 SNPs in 989 married couples. Panel 2: HRS data – genome-wide SNP-specific correlation for each of the 66,526 SNPs in 3,474 married couples (these 66,525 SNPs also available in FHS). The correlation was estimated using the mixed models with AR(1) covariance structure, controlling for population admixture.

Panel 1 of Figure 7 plots the genome-wide SNP-specific correlation for each of the 287,294 SNPs in 989 married couples in FHS. The correlation was estimated using the mixed model that allows positive and negative correlations. A large majority of the SNP correlations are scattered around 0 with a range of −0.10–0.10. Panel 2 of Figure 7 parallels Panel 1 of Figure 7 except it is based on HRS with a much larger sample of 3,474 married couples. The large sample explains the much narrower ranges of estimates of SNP correlations for HRS, ranging mostly between −0.05 and 0.05.

**Figure 8:**
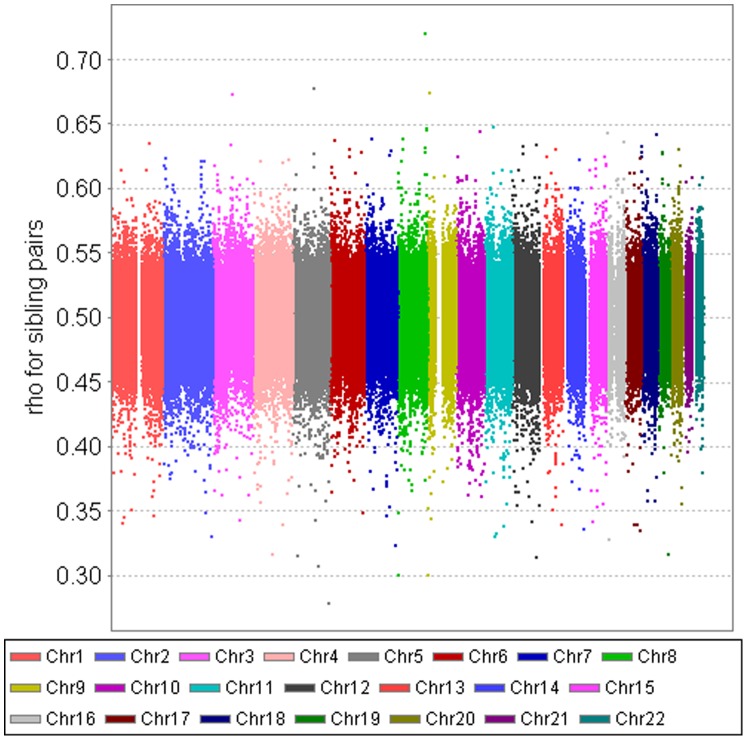
FHS data – genome-wide SNP-specific correlation for each of the 287,294 SNPs in 5,747 full sibling pairs. Both same-sex and opposite-sex full sibling pairs are included. The correlation was estimated using the mixed models with AR(1) covariance structure, controlling for population admixture.

**Figure 9:**
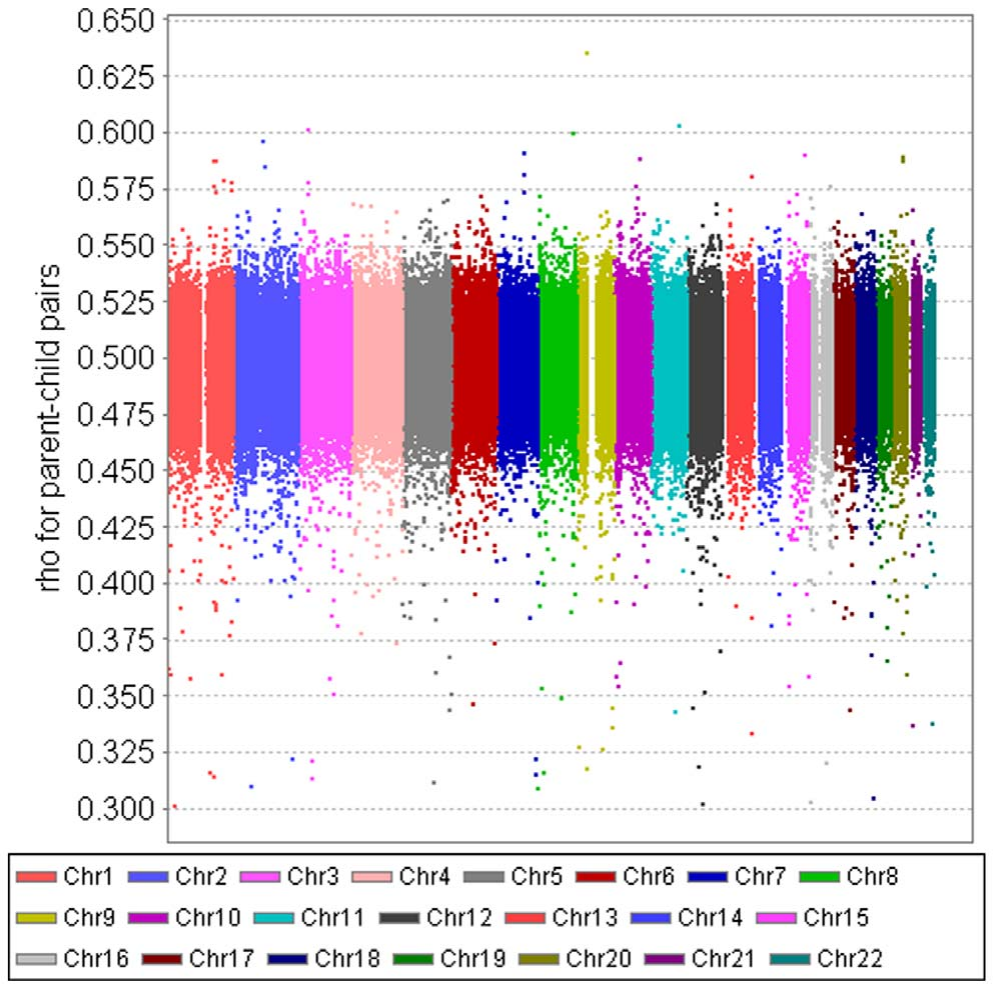
FHS data – genome-wide SNP-specific correlation for each of the 287,294 SNPs in 6,958 parent-child pairs. Both same-sex and opposite-sex parent-child pairs are included. The correlation was estimated using the mixed models with AR(1) covariance structure, controlling for population admixture.

To evaluate our measure of correlation, Figure 8 plots the genome-wide SNP correlation for each of the 287,294 SNPs in 5,713 full sibling pairs from FHS. Both same-sex and opposite-sex full sibling pairs are included. The large majority of the SNP correlations are scattered around 0.50 with a range of 0.40–0.60. Figure 9 presents the genome-wide SNP correlation for each of the 287,294 SNPs in 6,958 parent-child pairs. Again, both same-sex and opposite-sex parent-child pairs are included. The large majority of the SNP-specific correlations are scattered around 0.50 with a range of 0.45–0.55. As expected, the spread of the correlations for parent-child pairs is considerably narrower than that of full sibling pairs. The results in Figures 8 and 9 demonstrate that our method can produce the known patterns of genetic similarity in full sibling pairs and parent-child pairs.

Potentially problematic SNPs are those with a correlation estimate that is much less than 0.50 in the full-sibling analysis and the parent-child analysis. These SNPs do not affect our results of SNP correlations because each SNP correlation is independently calculated. In the calculation of the couple correlations where all SNPs were used in each regression, we excluded 231 out of the 287,525 SNPs. These excluded SNPs have either a full-sibling correlation less than 0.2 or greater than 0.8, or a parent-child correlation less than 0.3. The findings of couple correlations are not affected by whether these SNPs are included or excluded.

**Figure 10:**
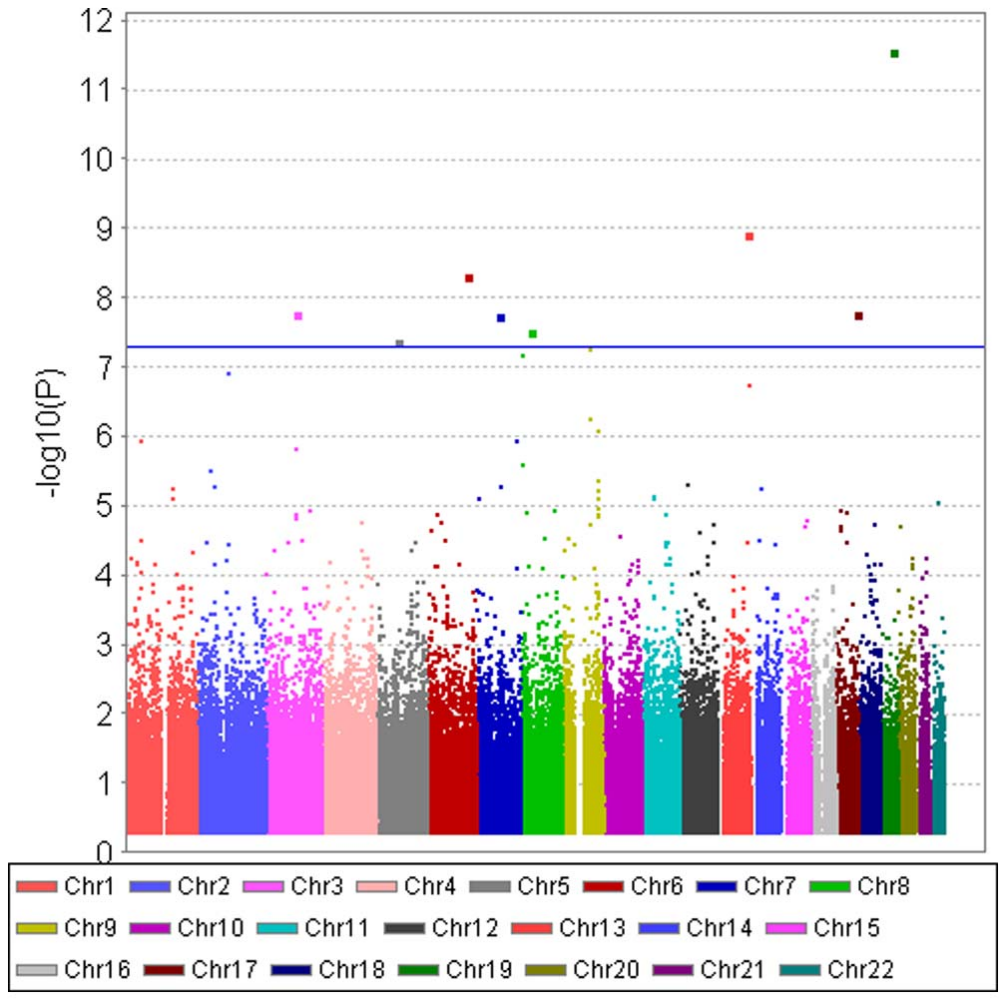
FHS data – the significance tests of SNP-specific correlations: the within-pair correlation of married couples against randomly-paired pairs. The tests for the 287,294 SNPs are shown in a Manhattan plot. The larger dots representing individual SNPs above the blue line indicate statistical significance at p<5×10^−8^.

**Figure 11:**
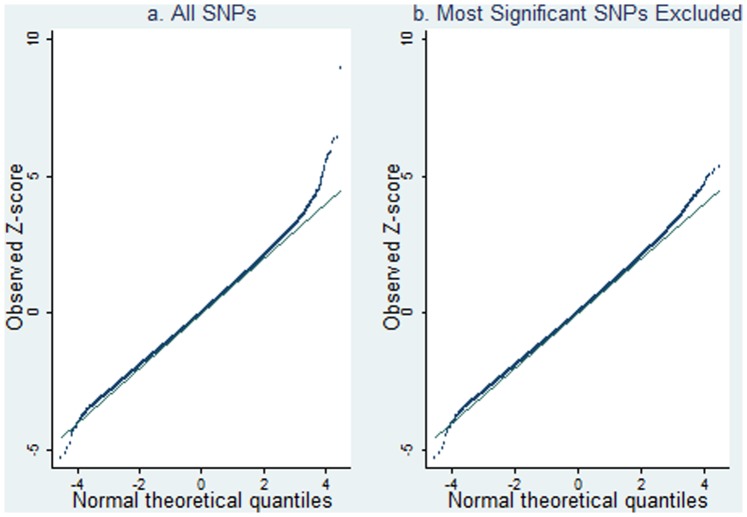
The QQ plot of observed Z-scores vs. expected Z-scores. The plot on the left side includes all 287,294 SNPs while the one on the right side excludes 8 SNPs with p-values smaller than 5×10^−8^.

Figure 10 shows the FHS permutation tests for the SNP-specific correlations in married couples against random pairs. As will be shown in Table 3, a small number of SNPs achieve a genome-wide significance with a p-value of 5×10^−8^ or smaller. The Q–Q plot of p-values from the SNP-specific correlations is presented in Figure 11, showing that some signals remain after removing the SNPs that have genome-wide significance (Panel 2 of Figure 11).

**Table 3:** FHS data – ten SNPs with the smallest p-values for the SNP-specific correlation in 989 married couples out of the 287,294 SNPs, with the correlation estimated from the mixed model after controlling for population admixture.

SNP	Chromosome(position)		Gene	Location	ReferenceAllele(Freq.)		MarriedPairs		SiblingPairs		Parent-childPairs	
							corr	*P*	corr	*P*	corr	*P*
rs16974794	19	(41499094)	CYP2B6	Intronic	A	(0.922)	0.27	0	0.47	0	0.35	0
rs1021652	17	(71403890)	SDK2	WNCG	A	(0.089)	0.20	9.30E-11	0.34	0	0.49	0
rs951954	2	(110459505)		Intergenic	T	(0.189)	0.20	1.11E-10	0.56	0	0.61	0
rs3007246	13	(105822046)		Intergenic	C	(0.928)	0.19	2.56E-10	0.56	0	0.55	0
rs16871467	5	(73484420)		Intergenic	G	(0.968)	0.18	2.43E-09	0.50	0	0.54	0
rs352416	8	(28452799)		Intergenic	C	(0.865)	0.17	9.45E-09	0.50	0	0.55	0
rs9483869	6	(136022914)	LINC00271	NoncodingRNA	T	(0.904)	0.17	1.59E-08	0.45	0	0.51	0
rs4449354	3	(105998897)		Intergenic	T	(0.090)	0.17	1.82E-08	0.49	0	0.52	0
rs16852244	3	(105993897)		Intergenic	T	(0.961)	0.16	5.15E-08	0.48	0	0.52	0
rs17155256	7	(81238711)	AY927633	WNCG	A	(0.843)	0.16	9.14E-08	0.46	0	0.53	0

Information is provided on SNP name, chromosome position, gene name when available, gene location, reference allele frequency, SNP correlation for married couples and p value from the permutation tests, correlation for full sibling pairs and p value, and correlation for parent-child pairs and p value.

WNCG: within noncoding gene&intronic.

NMDT: NMD transcript&intronic.

Table 3 lists 10 SNPs with the smallest p-values for the SNP-specific correlations in 989 married couples out of the 287,294 SNPs from FHS. The table lists SNP name, chromosome position, gene name when available, gene location, reference allele frequency, SNP correlation for married couples and p value from the permutation test, correlation for full sibling pairs and p value, and correlation for parent-child pairs and p value. Eight SNPs have a p-value 5×10^−8^ or smaller. The largest ten correlations are all positive. The SNP correlations from full-sibling pairs and parent-child pairs are in the expected ranges.

Our replication of the top ten SNPs from FHS (Table 3) using HRS yielded two SNPs (rs16871467 and rs9483869) that are statistically significant at 0.057 and 0.050, respectively. The correlations of these two SNPs are also positive, but smaller (0.026 and 0.027, respectively) than those in FHS. Overall, three of the SNPs in the HRS analysis with 66,526 SNPs achieve a genome-wide significance with a p-value of 5×10^−8^ or smaller.

Our final analysis is an FHS-66,526-SNP analysis for couple correlation. Panel 3 of Table 2 provides the observed distribution of within-pair allelic combination for different types of pairs for these SNPs in FHS. The table indicates that the distribution is much closer to the FHS distribution based on the full set of 287,294 SNPs with the same set of individuals than that in HRS based on the exactly the same set of SNPs but a different set of individuals. The regression analysis of couple correlation of these 66,526 SNPs in FHS confirm the findings from Panel 3 of Table 2 (not shown), providing evidence that married couple correlations are predominantly determined by individuals rather than SNPs and that the HRS 66,526-SNP analysis is likely generalizable to the full-SNP analysis.

## Discussion

In FHS, the two estimates of genome-wide couple correlation are 0.0018 (p = 3×10^−5^) and 0.oo18 (p = 10^−4^). These couple correlation estimates in HRS are 0.0016 (p = 8.29×10^−12^) and 0.0017 (p = 7.13×10^−13^). The much smaller p values from HRS in these estimates as well as other estimates are likely due to the much larger samples of HRS (3,474 couples) than FHS (989 couples). These estimates of couple correlations are not threatened by multiple testing.

Consistent with the estimates of Domingue et al [24], we show positive overall similarity in genomic assortment in married couples; however, our estimates seem much smaller than theirs (0.0016–0.0018 vs. 0.02–0.045). This is the case after taking into account that the two sets of estimates are not exactly comparable. As demonstrated in this analysis (Figures 2, 8, and 9), our estimates are essentially coefficients of genetic relatedness (*r*) and their estimates are quartile-transformed coefficients of kinship (*F*) with *r* = 2*F*, where *F* is untransformed coefficient of kinship. Our estimates in spousal correlation of educational attainment or years of education with standardization within each sex are 0.59 and 0.52 for HRS and FHS, respectively. One fifth to one third of these quantities are much larger than our estimated genome-wide couple correlation of 0.0016–0.0018. The variation in couple correlation across racial/ethnic groups is examined only in HRS. Less than 1% of the couples in FHS are ethnic minorities. In HRS, constraining the sample to non-Hispanic whites yields a somewhat smaller and statistically significant couple correlation of 0.0012.

The negative couple correlations in FHS are small and statistically non-significant (−.00008, p = .41;.00036, p = .18). The negative marital correlations in HRS are small and statistically significant (−0.0012, p = .0023; −0.0012, p = .0016). The positive couple correlations are much larger than negative correlations in absolute values in both FHS (0.001, p = .0043; 0.0012, p = .0088) and HRS (0.015, p = 1.66×10^−24^; 0.020, p = 7.75×10^−41^). The sizes of these estimates in FHS and HRS are consistent with what are suggested by the distribution of the allelic combination in Panels 1 and 2 of Table 2. The data in Table 2 can be considered findings that are more closely based on raw data than those from regression analysis. In both FHS and HRS, the positive correlation is much larger and more statistically significant than the negative correlation suggesting that genetic assortative mating is primarily positive.

For the analysis of SNP-specific correlation based on FHS, of the 287,294 SNP correlations, eight have a p-value 5×10^−8^ or smaller. These SNPs are all positively correlated between married couples, with a range of 0.16–0.27. We repeated the analysis of SNP correlations for these eight SNPs using HRS data. In HRS, two of these eight SNPs (rs9483869 and rs16871467) are statistically significant at about 0.05 and also correlated positively. However, these replications are suggestive rather than definitive because the two correlations in HRS are considerably smaller than those in FHS.

Neither rs9483869 nor rs16871467 has itself been identified as a statistically significant association in any previous GWAS analysis [25]. Rs9483869 is within an ncRNA called LINC00271, which is expressed in the brain [26]. Another SNP within LINC00271 (rs9494266) has been found to be a statistically significant hit in a GWAS on type 2 diabetes [27]. LINC00271 is in a region of high LD with the immediately adjacent gene AHI1, a gene involved in neurodevelopment and implicated in schizophrenia [27], [28]. Rs16871467 is approximately 246 kb downstream of ARHGF28, a member of the Rho guanine nucleotide exchange factor family. This protein interacts with low molecular weight neurofilament mRNA and may be involved in the formation of amyotrophic lateral sclerosis neurofilament aggregates [29]. Opposite, towards the chr5 telomere, the closest defined element is the retrogene C17orf76 antisense RNA 1, approximately 36 kb away. This SNP does reside in a DNAse I hypersensitive site defined by the ENCODE project [30], [31].

Genomic assortment in human marriages may vary over a number of factors. Different couples may assort on entirely different phenotypes and thus different genetic variants, which is expected to decrease the power of detecting SNP-specific correlations among couples. Genomic assortment may also be influenced by social and cultural contexts that vary across historical periods and geographic locations. American marriage is considerably different from marriage in other Western countries [32], not to mention marriage in non-Western countries. Pawlowski et al. [33] report an effect of World War II on mate preference in height. The advantage of taller males in the marriage market is evident among individuals born in the 1940 s, 1950 s and 1960 s, but not in the 1930 s. The authors suggest that this may be due to the relative scarcity of young men immediately after WWII. The genomic assortment may vary across geographic regions within the United States.

Overall, our data suggest a degree of genomic assortative mating at the allelic level in married couples who were born in the first half of the 20^th^ century in the United States. Apparently, this degree of genetic assortment averaged over the human genome is much smaller than the 0.20 Pearson had conjectured based on the observed correlations in height and arm span between husband and wife. As alluded earlier, certain genetic variants such as those underlying height are likely to be heavily assorted; however, the level of overall assortment in the genome seems much less.

However, a genomic correlation of 0.015–0.02 with married couples, estimated for the “positive” assorting SNPs in HRS, can represent an important genomic assortment for at least two reasons. A married-couple correlation may be compared with genetic relatedness among biological relatives. A genomic correlation of 0.015–0.02 is close to the average genomic correlation (0.0312) among second cousins (or the genomic correlation [0.0312] of an individual with his grandfather’s grandfather). While an individual passively and unselectively inherits half of his or her genes from each of the two parents, married individuals consciously or unconsciously assort on genes that play a strategic role in their reproductive marriages.

Our analysis of HRS reports a small but statistically significant negative genomic assortment, suggesting that negative genomic may, indeed, exist. This negative assortment contrasts conspicuously with the only-positive assortment among genetic relatives (see Figures 2 and 4).

Our interest is in assortative mating rather than genomic similarity related to population stratification and marriages between distant relatives. The principal components included in the analysis are effective (Figure 3); nevertheless, it might be difficult to differentiate low-level genomic similarity due to assortative mating from low-level genetic similarity due to distant genetic relatives marrying each other.

There is one important methodological limitation in the current analysis. As Wright [34] pointed out decades ago, assortative mating can only be done through external phenotypes and the same phenotype may result from different DNA sequences or non-homologous genes. For example, a married couple may assort by body weight, but the body weight of the husband and the wife may depend on different sets of genes (e.g., *FTO* vs *MC4R*). Such cases of genetic assortment are missed by direct allelic comparison between homologous genes, an approach used in this analysis.

The methodological limitation underestimates a more general form of genomic assortment, in which different allelic forms cause the same phenotype within the same gene or different genes. Assortative mating may actually occur at a higher level than we estimated in this project. Only when the general form of genomic assortment is taken into account could the impact of assortative mating suggested by Lewontin [3] and Thiessen and Gregg [6] be adequately evaluated.

## Methods

The Framingham Heart Study (FHS) is a community-based, prospective, longitudinal study following three generations of participants: (i) the Original Cohort enrolled in 1948 (N = 5,209); (ii) the Offspring Cohort consist of the children of the Original Cohort and their spouses, who were enrolled in 1971 (N = 5,124); and (iii) the Generation Three Cohort consists of the grandchildren of the Original Cohort, who were enrolled in 2002 (N = 4,095). More information on FHS can be found online [22]. Our analysis uses the 1,978 individuals or 989 married couples whose genotype data are available. These individuals are predominantly of European origin. Less than 1% of FHS respondents were racial/ethnic minorities.

Of the 14,428 study subjects in FHS, a total of 9,237 consenting individuals have been genotyped including 4,986 women and 4,251 men. Genotyping for FHS participants was performed by Affymetrix (Santa Clara, CA, USA) using the Affymetrix 500K GeneChip array. The Y chromosome was not genotyped. The standard quality control filter is applied. Individuals with 5% or more missing genotype data are excluded from analysis. X chromosome SNPs, SNPs with a call rate ≤99% or a minor allele frequency ≤0.01 are also eliminated from analysis. The application of the quality control filter leaves 8,738 individuals with 287,525 SNPs from the 500K genotype data.

The Health and Retirement Survey (HRS), launched in 1992, is a longitudinal study, surveying more than 22,000 Americans over the age of 50 every two years and collecting information on labor force participation and health transitions. The HRS began collecting salivary DNA in 2006 and has approximately >13,000 such DNA samples stored in repository. The genotyping for HRS was completed using the Illumina HumanOmni2.5-4v1 array, which includes more than one million SNPs. A total of 12,857 samples were genotyped and passed CIDR’s quality control (QC) process. The HRS analysis used samples of 6,948 individuals or 3,474 married couples that have passed the QC. A total of 66,526 SNPs out of 287,525 SNPs used in FHS were also genotyped in HRS.

In all our analyses, the outcome variable is the dosage of minor alleles for a SNP, which is standardized with mean = 0 and SD = 1; a correlation coefficient is used to measure genetic similarity. A correlation coefficient has a range of −1 to 1 allowing measurement of positive as well as negative assortment, and was used widely in measuring phenotypic similarity in studies of assortative mating. Correlation coefficients based on dosages of minor alleles are essentially coefficients of genetic relatedness (*r*). Because a coefficient of genetic relatedness is the most widely-used measurement of genetic relatedness among genetic relatives, our findings of genetic assortment among married couples can be readily understood and compared with the well-known genetic relatedness among full siblings (*r* = 0.5) and identical twins (*r* = 1).

Both married-couple-specific correlation and SNP-specific correlation are estimated by the following mixed linear model [35]:
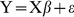
(1)where Y stands for standardized SNP dosage, *X* is a matrix of observed variables such as those used for controlling for population admixture, *β* is a coefficient vector of X including a standard intercept, and 
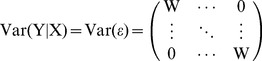
 with 
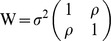
 in which *ρ* is either a couple correlation or a SNP correlation, depending on input data in Y. Model (1) is a special case of the auto-regressive AR(1) model. This AR(1) model allows for both positive and negative correlations, which correspond to positive and negative marital assortment.

For the couple correlation, Y_ij_ in Y is the SNP dosage for individual *i* and SNP *j* where *i = 1,2* indexing husband and wife in a married couple and *j* = 1,…,287,294 indexing the SNPs for FHS. Note that in the calculation for the couple correlation, the input data for a single mixed model FHS are a vector of SNP dosage with an extremely large dimension of 287,294×2 = 574,588. This dimension exceeds 2,000,000 if the entire set of HRS genome-wide genotype data are used for couple correlation analysis. For the SNP correlation, Y_ij_ in Y is the SNP dosage for individual *i* and married couple *j* where *i = 1,2* indexing husband and wife in a married couple and *j* = 1,…,989 indexing married couples for FHS. The mixed models for both couple correlations and SNP correlations were implemented in SAS [36].

More intuitively, our mixed model is analogous to a multilevel model in which IQ measures of students are clustered into schools [37]. IQ measures would be equivalent to SNP dosages and schools would be equivalent to couples. In FHS, each SNP-correlation regression model estimates the correlation of a SNP averaged over 989 couples, which is equivalent to a multilevel model that estimates the intra-class or within-school correlation of an IQ measure averaged over the schools in the analysis sample. The analogy may also be applied to our couple-correlation regression where the multilevel model analyzes only one school on a large number of different cognitive measures. The multilevel model would estimate a within-school correlation averaged over the large number of cognitive measures. The model can be identified because of multiple measures of cognitive outcomes. The model makes sense because we estimate an average genomic correlation within a couple, which is similar to genomic correlation within a pair of biological siblings. In FHS, our mixed couple-correlation model estimates a correlation within a couple averaged over 287,294 SNPs. In FHS, 989 couples yielded 989 such couple estimates.

To verify that our estimated correlation coefficients are essentially coefficients of genetic relatedness, the couple correlation and SNP correlation were also performed on 5,713 pairs of full siblings and 6,958 parent-child pairs. For full-sibling pairs, each couple correlation is based on all SNPs for a single full-sibling pair and each SNP correlation is based on all sibling pairs. The parent-child estimates parallel those of full-sibling pairs. The known genetic relatedness in full siblings and parent-children can be used as a benchmark against which the genetic similarity estimates from married couples can be evaluated. The SNP correlation based on full sibling pairs and parent-child pairs can also be used to check the quality of individual SNPs. If the sibling and parent-child correlation for a specific SNP deviate severely from what is expected, the quality of that particular SNP may be questioned.

To remove the effects of race and ethnicity on genomic assortment, principal components (PCs) were estimated in FHS and in HRS by Eigensoft [38], [39] and then included in regression analysis of couple and SNP correlations. Since principle components are influenced by correlation data, we excluded some of the correlated SNPs and correlated individuals when constructing PCs. To remove correlated SNPs, we used Plink to run LD-based SNP pruning and only kept the SNPs with pair-wise r^2^<0.2. To remove the correlated individuals, we used Plink to get the pairwise identity-by-descent (IBD) estimates, and kept those with estimated genome-wide pair-wise IBD <0.1. The PCs for the subjects that were excluded for the construction of PCs were subsequently calculated using the parameter coefficients obtained from those included in the PC estimation. For both FHS and HRS, seven largest PCs were used. Previous work shows that adjusting a small number of PCs is usually sufficient to account for population admixture [38]. For FHS, 92,648 SNPs were used to construct the PCs; for HRS, the PCs were constructed on the basis of the 67,385 SNPs.

Our mixed-model approach allows controlling population stratification in the regression analysis. For the SNP correlation, the seven largest PCs were included in Equation (1) as individual predictors. For the couple correlation, the seven largest PCs were used in a regression to predict the minor allele dosage of each SNP; the resulting residuals were then used as the outcome variable in Equation (1).

The statistical significance tests for couple correlations and SNP correlations are performed following the same principles in FHS and HRS. The couple correlations are evaluated via two permutation tests. Two permutation tests based on two quite different populations provide a robustness check for the results of significance tests. For FHS, the first permutation test is based on the individuals in the 989 married couples. We obtained 246,870 random pairs from these individuals who are genetically unrelated, unmarried, of the opposite sex, and with the male no more than 5 years older and no more than 2 years younger than the female. In the second permutation test based on all FHS individuals, we first randomly select a subset of 200,000 pairs from about 20 million possible unrelated opposite-sex pairs in FHS. A subset is selected to reduce computation. In both permutation tests, we (1) compute couple correlations for all these married couples and random pairs, (2) randomly draw 5,000 samples (N = 989) from the large pool of 200,000 (or 246,870) pairs without replacement, (3) randomly draw 5,000 samples (N = 989) from married couples with replacement, and (4) compare each of the 5,000 bootstrapped samples of married couples with the 5,000 random-pair samples using a t test.

A potential limitation of a couple correlation is that the positive and negative assortment within each married couple may cancel each other. To address this issue, we calculate two correlations for each couple, one using about half of the SNPs that contribute to the more “positive” assortment and the other using the half of SNPs that contribute to the more “negative” assortment.

The division of the entire set of the SNPs into “positive” and “negative” groups is based on the combination of minor allele dosage at each SNP for each couple. We use “02” to indicate that the minor allele dosage for a particular SNP for one spouse is “0” and for the other is “2”. The combination can only take one of the six forms: 02 or 20, 01 or 10, 11, 00, 12 or 21, and 22, where 0, 1 and 2 represent a minor allele dosage. A simulation based on the observed distribution of these combinations in the married couples of FHS yields an order of 02 or 20, 01 or 10, 11, 00, 12 or 21, and 22 according to how positive a contribution each of the six combinations makes to the overall couple correlation. These simulated results were used to order the SNPs in each couple dataset.

To provide more information on the simulation, we simulated paired data with six possible combinations of 02 or 20, 01 or 10, 11, 00, 12 or 21, and 22, assuming the distribution of each combination is the same as that in the observed genome-wide genotype data. We then compared each pair of the combinations with respect to their contributions to the overall correlation. For example, when comparing the contributions of 11 and 22, we assessed the change in the overall correlation as a response to increasing the proportion of 22 and reducing the proportion of 11, while keeping the same the proportions of other combinations. Comparing all possible pairs found that increasing the proportions of 00, 12 or 21, and 22 results in an increase of the overall correlation, whereas an increase in the proportions of 20 or 02, 10 or 01, and 11 results in a decrease of the overall correlation.

For each couple, the SNPs with the combinations of 20 or 02, 10 or 01, and 11 are included in the negative group and the SNPs with the combinations of 00, 12 or 21, and 22 are included in the positive group. The statistical tests for these positive and negative correlations are performed in a similar fashion as those for the overall couple correlation.

A Z-test and its associated p-value were obtained for each SNP correlation in both FHS and HRS. For FHS, each test is a comparison of the SNP correlation based on 989 married couples against the distribution of the same-SNP correlation calculated from the 5,000 samples of randomly paired opposite-sex pairs based on the entire FHS sample. Each of the 5,000 samples has a sample size of 989 pairs.

To summarize, this study consists of three parts. The first part is an FHS analysis; it uses all available SNPs (287,294) in FHS for both couple-correlation and SNP-correlation analysis. Part-2 is an HRS analysis. Part-2 SNP-correlation analysis only uses the 10 SNPs in HRS that have the smallest P-values in FHS; and part-2 couple-correlation analysis uses 66,526 SNPs in HRS that are also available in FHS. These SNPs are the only SNPs available in both FHS and HRS. Using exactly the same set of SNPs from two independent studies offers an opportunity to replicate the findings. A non-trivial reason for not using all SNPs available in HRS in couple-correlation analysis is computational. The analysis would have to estimate an extremely large number of mixed models for permutation tests, each model using a dataset with 2×2,000,000 = 4,000,000 rows of data. Part-3 analysis is a couple-correlation analysis using the 66,526 SNPs in FHS that are available in HRS. Thus, this part-3 FHS analysis uses exactly the same set of the 66,526 SNPs that the HRS analysis of couple correlation used, but a different set of individuals in FHS to calculate couple correlations. Comparing the findings from the FHS 287,294-SNP analysis and the FHS 66,526-SNP analysis provides evidence whether the findings from the 66,526-SNP analysis in HRS can be generalized to those of the 2,000,000-SNP analysis in HRS.
